# Exploring the etiology of dilated cardiomyopathy using Mendelian randomization

**DOI:** 10.3389/fcvm.2024.1364126

**Published:** 2024-08-26

**Authors:** SiYang Xue, HongJu Jiang

**Affiliations:** Department of Cardiology, The Second Affiliated Hospital of Shandong University of Traditional Chinese Medicine, Jinan, Shandong, China

**Keywords:** Mendelian randomization, dilated cardiomyopathy (DCM), genome-wide association study (GWAS), etiology, single nucleotide polymorphism (SNP)

## Abstract

**Background:**

Observational clinical studies suggest an association between dilated cardiomyopathy (DCM) and various factors including titin, cardiac troponin I (CTnI), desmocollin-2, the perinatal period, alcoholism, Behçet's disease, systemic lupus erythematosus, hyperthyroidism and thyrotoxicosis, hypothyroidism, carnitine metabolic disorder, and renal insufficiency. The causal nature of these associations remains uncertain. This study aims to explore these correlations using the Mendelian randomization (MR) approach.

**Objective:**

To investigate the etiology of DCM through Mendelian randomization analysis.

**Methods:**

Data mining was conducted in genome-wide association study databases, focusing on variant target proteins (titin, CTnI, desmocollin-2), the perinatal period, alcoholism, Behçet's disease, systemic lupus erythematosus, hyperthyroidism and thyrotoxicosis, hypothyroidism, carnitine metabolic disorder, and renal insufficiency, with DCM as the outcome. The analysis employed various regression models, namely, the inverse-variance weighted (IVW), MR-Egger, simple mode, weighted median, and weighted mode methods.

**Results:**

The IVW results showed a correlation between titin protein and DCM, identifying titin as a protective factor [OR = 0.856, 95% CI (0.744–0.985), *P *= 0.030]. CTnI protein correlated with DCM, marking it as a risk factor [OR = 1.204, 95% CI (1.010–1.436), *P *= 0.040]. Desmocollin-2 also correlated with DCM and was recognized as a risk factor [OR = 1.309, 95% CI (1.085–1.579), *P *= 0.005]. However, no causal relationship was found between the perinatal period, alcoholism, Behçet's disease, systemic lupus erythematosus, hyperthyroidism and thyrotoxicosis, hypothyroidism, carnitine metabolic disorder, renal insufficiency, and DCM (*P *> 0.05). The MR-Egger intercept test indicated no pleiotropy (*P *> 0.05), affirming the effectiveness of Mendelian randomization in causal inference.

**Conclusion:**

Titin, CTnI, and desmocollin-2 proteins were identified as independent risk factors for DCM. Contrasting with previous observational studies, no causal relationship was observed between DCM and the perinatal period, alcoholism, Behçet's disease, systemic lupus erythematosus, hyperthyroidism and thyrotoxicosis, hypothyroidism, carnitine metabolic disorder, or renal insufficiency.

## 1 Introduction

Dilated cardiomyopathy (DCM) is a common disease that leads to heart failure, arrhythmias, and sudden cardiac death. It is the most prevalent form of cardiomyopathy globally, affecting individuals of all ages and genders (1). Since 2013, the prevalence of DCM has risen from 1/2,500 to 1/250 (2). Associated with cardiogenic sudden death and heart failure, DCM results in high hospital admission rates and a potential need for heart transplantation, causing considerable suffering and financial burdens for patients.

Despite its global public health impact, no effective cures for DCM exist. Treatment focuses on relieving heart failure symptoms, improving myocardial remodeling, and even implantation of LVADs or heart transplants in the terminal stage (3).The lack of a comprehensive understanding of DCM's pathogenesis and risk factors contributes to this challenge. Previous studies have reported several risk factors for DCM (4, 5), including functional variants in target proteins [titin, cardiac troponin I (CTnI), and desmocollin-2]; the perinatal period; alcoholism; secondary cardiomyopathy due to autoimmune diseases [Behçet's disease and systemic lupus erythematosus (SLE)]; metabolic, endocrine, and nutritional diseases (hyperthyroidism and thyrotoxicosis, hypothyroidism, and carnitine metabolic disorder); and cardiomyopathy secondary to other organ diseases (uremia). The etiology of DCM is complex and multifaceted. The cure rate of DCM based on risk factors is not very satisfactory (4, 5). Genetic studies can provide new ideas to further clarify the etiology of DCM. Therefore, understanding DCM's etiology is vital. Mendelian randomization (MR) uses genetic variations as instrumental variables (IVs) (6), offering an approach akin to randomized controlled trials (7). This statistical model, free from the confounding and reverse causation biases of observational studies, assesses the causal relationship between exposure and outcome factors (8). Our aim was to demonstrate a causal relationship between DCM and related causes using Mendelian randomization.

## 2 Materials and methods

### 2.1 Study design

**Figure 1:**
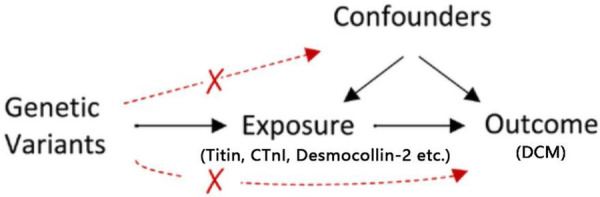
The schematic illustration of a two-sample MR study.

MR analysis adheres to three key assumptions: (1) the relevance assumption—genetic IVs should be closely associated with exposure factors; (2) the independence assumption—genetic IVs should not be linked with potential confounders; and (3) the exclusion restriction assumption—genetic IVs should influence the outcome solely through exposure factors (Figure 1). This study uses MR to examine the causal relationship between existing etiologies and DCM, employing variant target proteins (titin, CTnI, and desmocollin-2), perinatal period, alcoholism, Behçet's disease, systemic lupus erythematosus, hyperthyroidism and thyrotoxicosis, hypothyroidism, carnitine metabolic disorder, and renal insufficiency as exposure factors, and DCM as the outcome factor.

### 2.2 Data sources

We obtained genetic data for exposure and outcome factors from genome-wide association studies (GWAS). Genetic data for titin, CTnI, and desmocollin-2 came from the study by Sun et al. (9), comprising 3,301 samples and 10,534,735 single nucleotide polymorphisms (SNPs); perinatal period genetic data from the Integrative Epidemiology Unit (IEU) Open GWAS database, with 218,792 samples and 16,380,466 SNPs; alcoholism genetic data from the IEU Open GWAS database, encompassing 217,277 samples and 16,380,440 SNPs; lupus genetic data from the study by Sakaue et al. (10), featuring 482,911 samples and 24,198,877 SNPs; Behçet's disease genetic data also from Sakaue et al. (10), including 317,252 samples and 19,084,009 SNPs; hyperthyroidism and thyrotoxicosis genetic data from Dönertaş et al. (11), with 484,598 samples and 9,587,836 SNPs; hypothyroidism genetic data again from Sakaue et al. (10), comprising 410,141 samples and 24,138,872 SNPs; carnitine metabolic disorder genetic data from Shin et al. (12), including 7,797 samples and 2,545,563 SNPs; and finally, renal insufficiency genetic data from Sakaue et al. (10), featuring 482,858 samples and 24,185,976 SNPs. Genetic data for the outcome factor, DCM, were obtained from Sakaue et al. (10), encompassing 355,381 samples and 19,080,278 SNPs (Table 1).

**Table 1:** Relevant data extracted from the GWAS database.

	Year	Population	Sample size	SNP count	GWAS ID
Exposure					
Titin protein	2018	European	3,301	10,534,735	prot-a-3116
CTnI	2018	European	3,301	10,534,735	prot-a-3066
Desmocollin-2	2018	European	3,301	10,534,735	prot-a-866
Perinatal period	2021	European	218,792	16,380,466	finn-b-P16_RESPI_CARDIOV_DISORD_SPE CIFIC_PERINA_PERIOD
Alcoholism	2022	European	217,277	16,380,440	finn-b-ST19_TOXIC_EFFECT_ALCOHOL
Lupus	2021	European	482,911	24,198,877	ebi-a-GCST90018917
Behçet's disease	2021	European	317,252	19,084,009	ebi-a-GCST90018798
Hyperthyroidism/thyrotoxicosis	2021	European	484,598	9,587,836	ebi-a-GCST90038636
Hypothyroidism	2021	European	410,141	24,138,872	ebi-a-GCST90018862
Carnitine metabolic disorder	2014	European	7,797	2,545,563	met-a-379
Renal insufficiency	2021	European	482,858	24,185,976	ebi-a-GCST90018822
Outcome					
DCM	2021	European	355,381	19,080,278	ebi-a-GCST90018834

### 2.3 Selection of instrumental variables

IVs for exposure factors must meet these criteria: (1) All IVs should be genome-wide significant (*P *< 5 × 10^−6^/*P *< 5 × 10^−8^); (2) Linkage disequilibrium parameter (*R*^2^) = 0.001, with a regional range within 10,000 kb; (3) The *F*-statistic assesses weak instrument bias, representing the strength of individual SNPs. An SNP is considered strong enough to avoid biases if its *F*-statistic is >10. The *F*-statistic formula is *F = β*^2^*/SE*^2^ (13). We calculated the *F*-statistic and excluded SNPs with *F* <10 to prevent bias; (4) Knock out any palindromic SNPs (14) or incompatible alleles; (5) SNPs linked to confounding factors are removed. This involves consulting the PhenoScanner database and excluding SNPs associated with exposure or outcomes. The refined SNPs were used as IVs in this study. This removed confounding factors including malnutrition, strenuous exercise, overexertion, and radiation exposure (4).

### 2.4 MR analysis

The primary analysis method in this study was the inverse-variance weighted (IVW) method, which offers robust results without pleiotropy. Supplementary methods included MR-Egger, simple mode, weighted median (WM), and weighted mode to verify the robustness of the main analysis. For sensitivity analyses, we used a leave-one-out analysis approach and the outlier detection (MR-PRESSO) method. The outlier method identifies and interprets significant outliers that may increase heterogeneity and horizontal pleiotropy. The leave-one-out sensitivity analysis examines the impact of individual SNPs on the causal relationship. Sensitivity analysis for each SNP helps exclude variables that may significantly affect the results. Pleiotropy was assessed via the MR-Egger intercept test; a *P* <0.05 indicates pleiotropy. Heterogeneity was evaluated using Cochran's Q-value from the IVW and MR-Egger tests, with Q-Pval <0.05 signaling heterogeneity. In cases of heterogeneity, a random-effects model is applied in MR analysis. We conducted MR analysis using the “TwoSampleMR,” “MRPRESSO,” and “forestploter” packages in R software (version 4.3.2, R Foundation, Vienna, Austria).

## 3 Results

### 3.1 Instrumental variable selection results

In this study, a complete GWAS database was used to screen and remove confounding factors, and SNPs with a strong association with exposure factors were obtained. To avoid the phenomenon of linkage disequilibrium and reduce the bias caused by weak instruments and other problems, screening was further carried out according to the filter conditions of the instrumental variables mentioned above. We further excluded any palindromic SNPs. A palindromic SNP is an SNP in which the possible alleles for that SNP are the same alleles that would pair with each other in the double helix structure. When this occurs, alleles on the forward strand are the same as on the reverse strand. In two-sample MR, it can be difficult to harmonize palindromic SNPs if it is unclear which way the alleles are presented in the data sources providing information on the IV-exposure association and the IV-outcome associations. Therefore, it is necessary to delete the palindromic SNPs to ensure the accuracy of the results (14). Finally, a total of 164 SNPs (Supplementary Table S1) were included, all with an *F*-statistic greater than 10, and the results of MR analysis were not affected by weak instrumental variables. According to the MR-Egger intercept test, none of the data had any pleiotropy (*P* > 0.05) and the MR analysis could be performed.

### 3.2 Univariate MR analysis

#### 3.2.1 Analysis of titin protein

**Figure 2:**
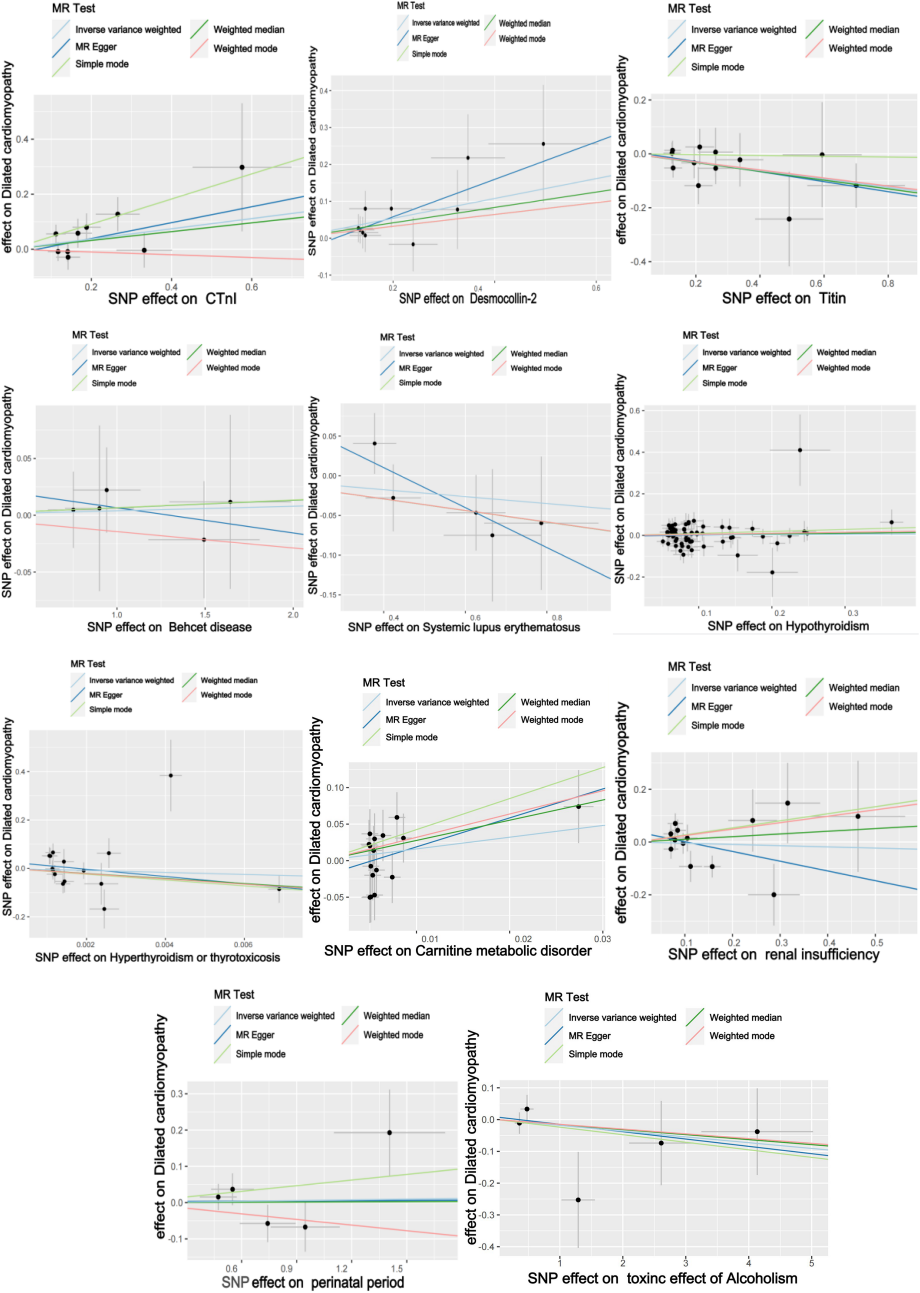
Univariate MR analysis scatter plots.

**Figure 3:**
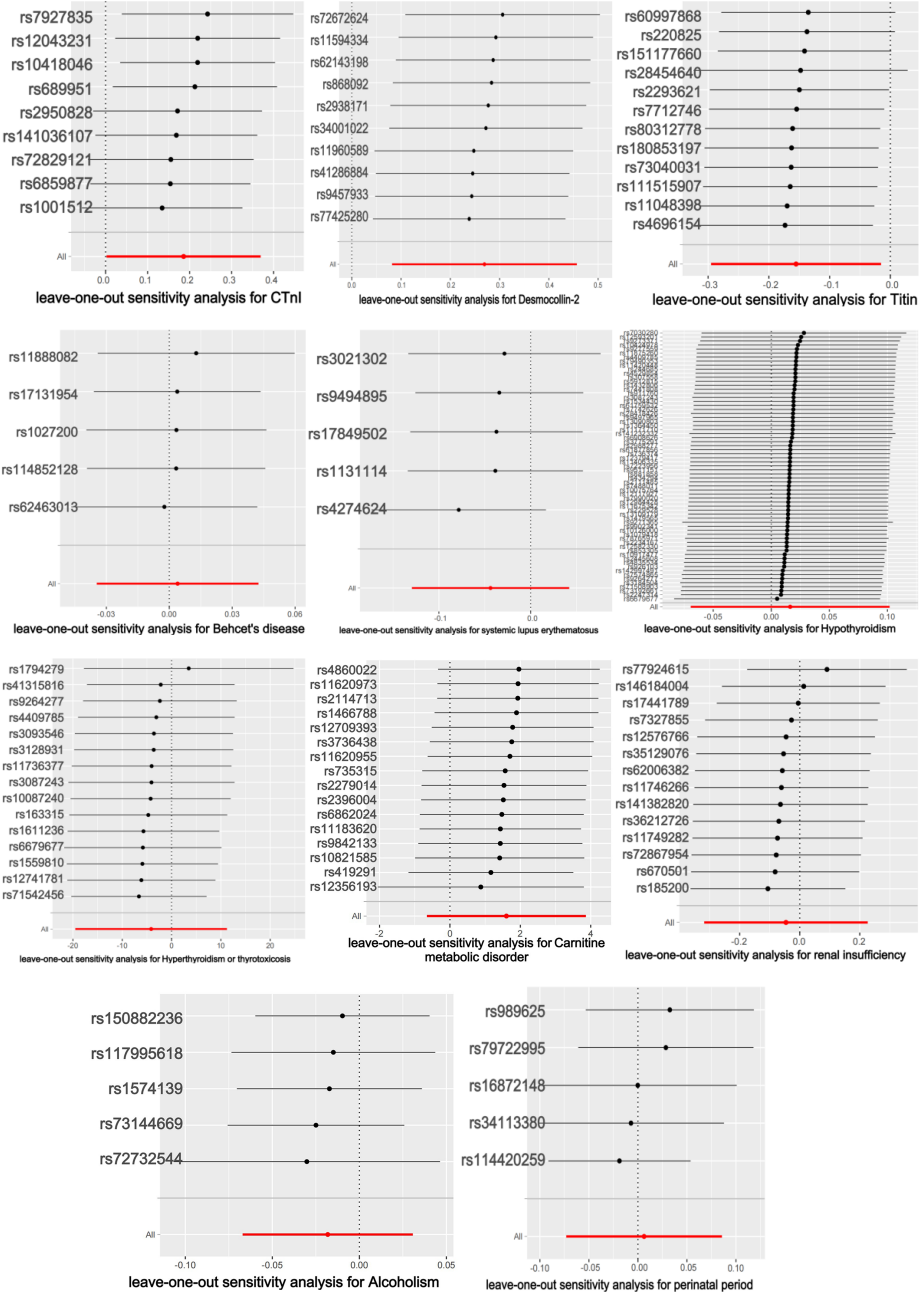
Results of the leave-one-out method.

The titin protein analysis showed a consistent direction across all five methods (Figure 2). Before MR analysis, palindromic SNP rs62127764 was removed. The heterogeneity test showed no heterogeneity (Q-Pval > 0.05), and a fixed-effects model IVW method was applied. Sensitivity analysis with leave-one-out analysis and the MR-PRESSO method identified no impactful SNPs on the causal relationship (Figure 3). The MR-Egger test indicated no pleiotropy (*P *= 0.783). The IVW results revealed a correlation between titin protein and DCM (Table 2), suggesting it as a protective factor [OR = 0.856, 95% CI (0.744–0.985), *P *= 0.030].

**Table 2:** Results of univariate Mendelian randomization analysis.

Exposure	Outcome	Analysis methods	SNPs (n)	*β*	*SE*	*P*	Adjusted *P*	OR	95% CI
Titin protein	DCM	IVW	12	−0.155	0.071	0.030	0.039	0.856	0.744–0.985
CTnI			9	0.186	0.090	0.040	0.039	1.204	1.010–1.436
Desmocollin-2			10	0.269	0.096	0.005	0.015	1.309	1.085–1.579
Perinatal period			5	0.006	0.041	0.876		1.006	0.929–1.090
Alcoholism			5	−0.018	0.025	0.467		0.982	0.935–1.031
Lupus			5	−0.044	0.044	0.317		0.957	0.879–1.043
Behçet's disease			5	0.004	0.020	0.840		1.004	0.966–1.043
Hyperthyroidism/thyrotoxicosis			15	−4.145	7.844	0.597		0.016	3.332 × 10^9^–7.538 × 10^4^
Hypothyroidism			67	0.016	0.044	0.710		1.016	0.933–1.107
Carnitine metabolic disorder			16	1.604	1.152	0.164		4.973	0.520–47.583
Renal insufficiency			14	−0.046	0.139	0.741		0.955	0.728–1.254

#### 3.2.2 Analysis of CTnI

**Figure 4:**
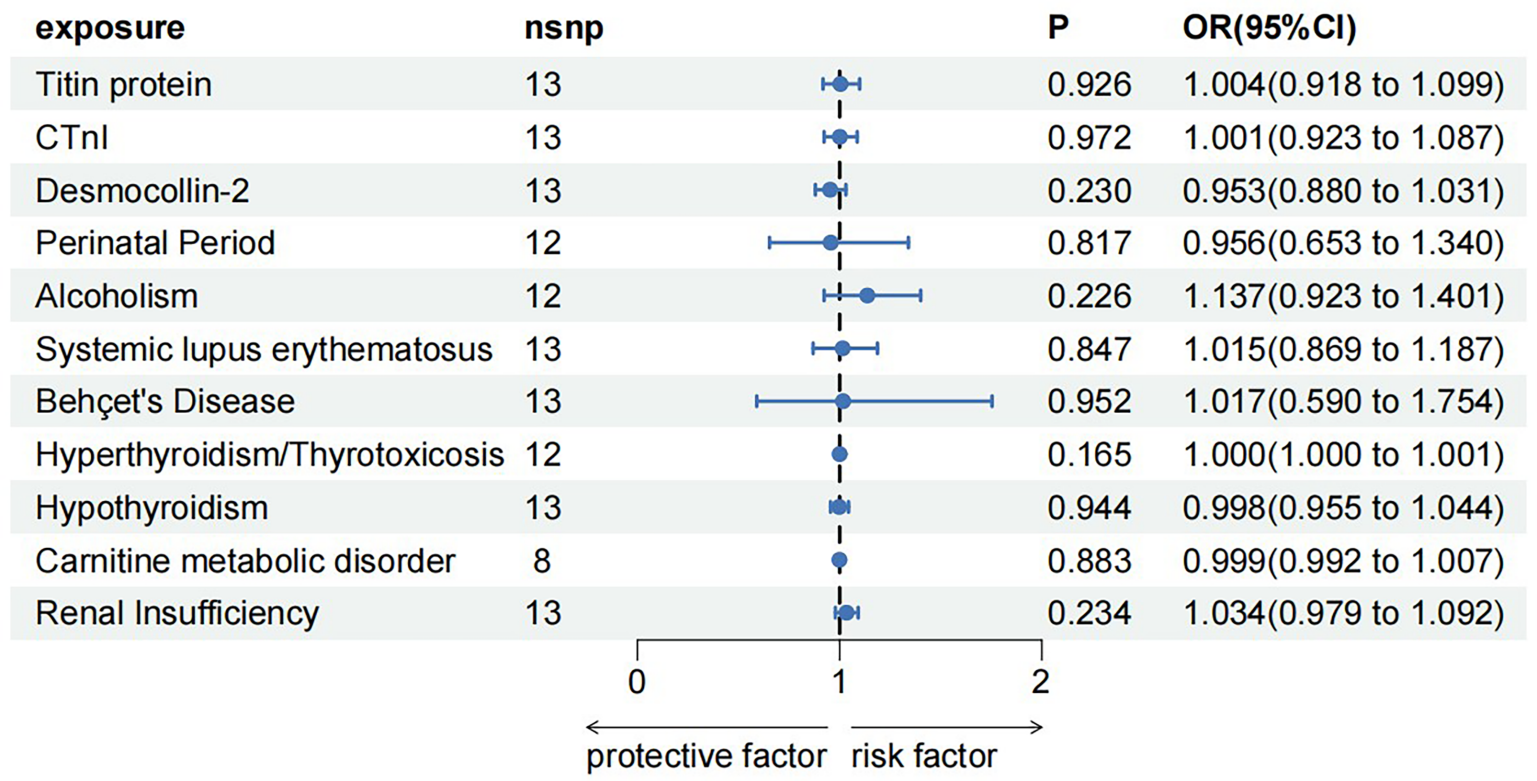
Results of the reverse Mendelian randomization analysis.

CTnI protein analysis displayed a consistent direction across all five methods (Figure 2). No palindromic SNPs were found prior to MR analysis. The heterogeneity test indicated no heterogeneity (Q-Pval > 0.05), utilizing a fixed-effects model IVW method. Sensitivity analysis with leave-one-out analysis and the MR-PRESSO method showed no significant SNPs affecting the causal relationship (Figure 3). The MR-Egger test revealed no pleiotropy (*P *= 0.680). The IVW results demonstrated a correlation between the CTnI protein and DCM (Figure 4), identifying it as a risk factor [OR = 1.204, 95% CI (1.009–1.436), *P *= 0.039].

#### 3.2.3 Analysis of desmocollin-2

Desmocollin-2 analysis indicated a consistent direction in all five methods (Figure 2). Palindromic SNPs rs147947012 and rs1789063 were excluded before MR analysis. The heterogeneity test showed no heterogeneity (Q-Pval > 0.05), leading to a fixed-effects model IVW approach. Sensitivity analysis with leave-one-out analysis and the MR-PRESSO method did not find any impactful SNPs on the causal relationship (Figure 3). The MR-Egger test indicated no pleiotropy (*P *= 0.396). The IVW results suggested desmocollin-2 was a risk factor for DCM (Table 2) [OR = 1.309, 95% CI (1.085–1.579), *P *= 0.005].

#### 3.2.4 Analysis of the perinatal period

The perinatal period analysis showed a consistent direction across all five methods (Figure 2). Before MR analysis, no palindromic SNPs were detected. The heterogeneity test indicated no heterogeneity (Q-Pval > 0.05), employing a fixed-effects model IVW method. Sensitivity analysis with leave-one-out analysis and the MR-PRESSO method identified no significant SNPs affecting the causal relationship (Figure 3). The MR-Egger test showed no pleiotropy (*P *= 0.977). The IVW results suggested the perinatal period was a risk factor for DCM (Table 2) [OR = 1.006, 95% CI (0.929–1.090), *P *= 0.876], but this was not statistically significant (*P *> 0.05).

#### 3.2.5 Analysis of alcoholism

Alcoholism analysis demonstrated consistent direction in all five methods (Figure 2). Before MR analysis, palindromic SNP rs57490218 was excluded. The heterogeneity test showed no heterogeneity (Q-Pval > 0.05), utilizing a fixed-effects model IVW method. Sensitivity analysis with leave-one-out analysis and MR-PRESSO method found no impactful SNPs on the causal relationship (Figure 3). MR-Egger test indicated no pleiotropy (*P *= 0.830). IVW results suggested alcoholism as a protective factor for DCM (Table 2) [OR = 0.982, 95% CI (0.935–1.031), *P *= 0.467], but this was not statistically significant (*P *> 0.05).

#### 3.2.6 Analysis of SLE

SLE analysis showed a consistent direction in all five methods (Figure 2). No palindromic SNPs were found before MR analysis. The heterogeneity test indicated no heterogeneity (Q-Pval > 0.05), applying a fixed-effects model IVW method. Sensitivity analysis with leave-one-out analysis and MR-PRESSO method did not reveal significant SNPs affecting the causal relationship (Figure 3). The MR-Egger test showed no pleiotropy (*P *= 0.286). The IVW results indicated SLE was a risk factor for DCM (Table 2) [OR = 0.957, 95% CI (0.879–1.043), *P *= 0.317], but this was not statistically significant (*P *> 0.05).

#### 3.2.7 Analysis of Behçet's disease

The analysis of Behçet's disease showed a consistent direction in three methods (Figure 2). Before MR analysis, palindromic SNP rs2697704 and weak instrumental variable rs72735844 with an *F* <10 were excluded. The heterogeneity test showed no heterogeneity (Q-Pval > 0.05), using a fixed-effects model IVW method. Sensitivity analysis with leave-one-out analysis and the MR-PRESSO method found no significant SNPs affecting the causal relationship (Figure 3). The MR-Egger test indicated no pleiotropy (*P *= 0.711). The IVW results suggested Behçet's disease was a risk factor for DCM (Table 2) [OR = 1.004, 95% CI (0.966–1.043), *P *= 0.840], but this was not statistically significant (*P *> 0.05).

#### 3.2.8 Analysis of hyperthyroidism and thyrotoxicosis

The analysis of hyperthyroidism and thyrotoxicosis showed a consistent direction in all five methods (Figure 2). Before MR analysis, palindromic SNPs rs12999008 and rs4903961 were excluded. The heterogeneity test indicated heterogeneity (Q-Pval < 0.05), and a random-effect model IVW method was applied. Sensitivity analysis with leave-one-out analysis and the MR-PRESSO method identified no significant SNPs on the causal relationship (Figure 3). The MR-Egger test showed no pleiotropy (*P *= 0.3324255). The IVW results indicated hyperthyroidism and thyrotoxicosis were protective factors for DCM (Table 2) [OR = 1.58 × 10^−2^, 95% CI (3.332 × 10^9^ to 7.538 × 10^4^), *P *= 0.597], but this was not statistically significant (*P *> 0.05).

#### 3.2.9 Analysis of hypothyroidism

The analysis of hypothyroidism showed a consistent direction across all five methods (Figure 2). Before MR analysis, palindromic SNPs rs1065386, rs114285740, rs187707293, rs2114702, rs2412976, rs2921053, rs3118469, and rs34536443, and incompatible allele rs11406335 were excluded. The heterogeneity test showed no heterogeneity (Q-Pval > 0.05), utilizing a fixed-effects model IVW method. Sensitivity analysis with leave-one-out analysis and the MR-PRESSO method revealed no significant SNPs impacting the causal relationship (Figure 3). The MR-Egger test indicated no pleiotropy (*P *= 0.755607). The IVW results suggested hypothyroidism was a risk factor for DCM (Table 2) [OR = 1.016, 95% CI (0.933–1.107), *P *= 0.710], but this was not statistically significant (*P *> 0.05).

#### 3.2.10 Analysis of carnitine metabolic disorder

The carnitine metabolic disorder analysis showed a consistent direction in all five methods (Figure 2). Before MR analysis, palindromic SNP rs13182512 was excluded. The heterogeneity test showed no heterogeneity (Q-Pval > 0.05), employing a fixed-effects model IVW method. Sensitivity analysis with leave-one-out analysis and the MR-PRESSO method identified no significant SNPs affecting the causal relationship (Figure 3). The MR-Egger test indicated no pleiotropy (*P *= 0.2428895). The IVW results suggested carnitine metabolic disorder was a risk factor for DCM (Table 2) [OR = 4.973, 95% CI (0.520–47.583), *P *= 0.164], but this was not statistically significant (*P *> 0.05).

#### 3.2.11 Analysis of renal insufficiency

The renal insufficiency analysis showed a consistent direction in two methods (Figure 2). Before MR analysis, palindromic SNPs rs11146948, rs12793321, rs3115665, rs4936552, and rs77638060 were excluded. The heterogeneity test indicated no heterogeneity (Q-Pval > 0.05), using a fixed-effects model IVW method. Sensitivity analysis with leave-one-out analysis and the MR-PRESSO method found no significant SNPs impacting the causal relationship (Figure 3). The MR-Egger test showed no pleiotropy (*P *= 0.279). The IVW results indicated renal insufficiency was a protective factor for DCM (Table 2) [OR = 0.955, 95% CI (0.728–1.254), *P *= 0.741], but this was not statistically significant (*P *> 0.05).

This study involved multiple exposures. To reduce the probability of a type I error, the FDR (Benjamini–Hochberg) method was used to appropriately correct the test *P*-value. The corrected results were 0.039, 0.039, and 0.015, respectively, and the FDR values were lower than 0.05, indicating significant differences among the three positive exposures.

### 3.3 Reverse MR analysis

To rule out a reverse causal relationship between DCM and a known etiology and meet the third key assumption of MR, two samples were used for univariate reverse MR analysis. The *F*-statistic for each SNP of the DCM was calculated to be >10 before MR analysis. Except for carnitine metabolic disorder, palindromic SNPs rs12051750 and rs62240972 were eliminated before MR analysis of the remaining 10 exposures (carnitine metabolic disorder had no palindromic SNPs). Heterogeneity tests showed no heterogeneity (Q-Pval > 0.05) using the fixed-effects model IVW method for analysis. Sensitivity analysis using the leave-one-out sensitivity analysis and the MR-PRESSO method did not identify SNPs that had a significant impact on the causal relationships. The MR-Egger test showed no pleiotropy, with *P*-values >0.05. The results of IVW method showed that DCM was not an influencing factor of known etiology and there is no causal relationship between them (Figure 4).

## 4 Discussion

In this study, we utilized the GWAS database to select SNPs closely related to DCM and its etiologies. We employed five methods (MR-Egger, weighted median, fixed-effects model IVW, random-effect Model IVW, simple mode, weighted mode) to analyze the causal relationships. Mutations in genes linked to DCM leading to functional abnormalities in the target proteins (titin, CTnI, and desmocollin-2) demonstrated a causal relationship with DCM. Our findings contrast with previous observational clinical studies, demonstrating that factors such as alcoholism, the perinatal period, lupus, Behçet's disease, hyperthyroidism, hypothyroidism, carnitine metabolic disorder, and renal insufficiency showed no causal relationship with DCM. Our MR analysis indicates that decreased titin levels and increased CTnI and desmocollin-2 levels heighten DCM risk, confirming a significant causal relationship and aligning with existing research (5, 15). Previous studies have suggested that primary DCM is an autosomal genetic disorder caused by gene mutations. In this study, MR was used to explore the etiology of DCM at the genetic level, which is of great significance for deepening the understanding of the etiology of DCM. Titin is an important sarcomere protein, the largest protein known to be expressed in the heart, and maintains the structural integrity and elasticity of myocardial cells, provides power for myocardium, and participates in the regulation of myocardial sarcomere contraction and signaling (16). It also plays an active role in myocardial contraction, diastole, and passive tension. Titin truncating variants (TTNtvs) represent the most common cause of familial and sporadic DCM (17).Over the years, a variety of hypotheses have been proposed to explain the pathogenicity of TTNtvs; however, the two most popular have been haploinsufficiency (18) and the poison peptide hypothesis (19). In the case of the former, titin production from the single unaffected allele is insufficient to maintain appropriate cardiac function, causing a decrease in the left ventricular ejection fraction. The latter posits that the truncated protein acts in a manner that disrupts normal heart function. The findings of recent studies indicate that both mechanisms contribute to the development of DCM in TTNtv carriers (20).The pathophysiological changes of DCM are mainly cardiomyocyte hypertrophy, fibrous tissue hyperplasia, and non-specific degenerative changes and interstitial fibrosis. The lesion is diffuse and affects the whole heart, but the left ventricle is mainly dilated. The hypertrophy of the ventricular wall is relatively insignificant or even thin, and the systolic function of the heart is reduced. Thus, the titin protein plays a protective role in DCM. This is in line with our findings. CTnI, the striated muscle troponin complex's inhibitory subunit, mediates muscle relaxation by preventing myosin-actin binding and regulates Ca^2+^ sensitivity (21, 22). Increased CTnI levels can reduce cardiac muscle cell contractility. Studies have shown a potential link between CTnI and the prognosis of patients with DCM. Elevated serum CTnI is the preferred marker for diagnosing myocardial injury due to its excellent sensitivity and specificity, and its elevated levels are an independent predictor of increased all-cause mortality in patients with DCM (23). Cardiac desmosomes are cell–cell junctions that connect the intercalated disc to the intermediate filament system, containing a total of five desmosomal proteins that have important mechanical and signaling functions. Desmocollin-2, as one of the five desmosomal transmembrane proteins in the heart, assembles a desmosome junction with the other four desmosomal proteins to ensure the structural and functional integrity of the myocardium. It is also involved in cell signaling, apoptosis, and cell-to-cell adhesion (24). Heart-specific overexpression of desmocollin-2 induces myocardial necrosis, acute inflammation, and patchy cardiac fibrotic remodeling, leading to fulminant cardiomyopathy with extensive cardiac fibrosis with necrosis and calcification, resulting in shortened ventricular fraction and reduced ejection fraction (25). Furthermore, mutations in the desmocollin-2 gene may cause cardiomyopathy and related arrhythmias (26, 27). These results suggest that CTnI and desmocollin-2 can damage cardiomyocytes and cause or aggravate DCM. These may explain our findings that CTnI and desmocollin-2 are risk factors for cardiopathy.

Previous observational clinical studies have suggested that cardiac disease is associated with alcoholism, perinatal period, lupus erythematosus, Behcet's disease, hyperthyroidism and thyrotoxicosis, hypothyroidism, carnitine metabolic disorder, and renal insufficiency, but this study ruled out a causal relationship with them using Mendelian randomization. The reasons may be related to the following: (1) MR analysis, based on the GWAS database, selects genetic variants (SNPs) as instrumental variables, effectively avoiding biases from lifestyle, social environment, and other confounding factors; (2) Previous studies have only included myocardial damage as the cause of cardiac disease, but have not further clarified whether there is a causal relationship between them; (3) This study employed large-sample MR analysis, offering more convincing results compared to previous small-sample observational studies.

Limitations include the following: (1) The GWAS data for the MR analysis population are from Europeans, and racial differences in allele frequencies and disease prevalence might affect the applicability of the findings; (2) GWAS database limitations mean detailed data such as disease severity is unavailable; (3) SNPs for hyperthyroidism, thyrotoxicosis, and carnitine metabolic disorder with an *F*-statistic of <10 were used and such weak instrumental variables could introduce bias; (4) The GWAS database lacks data on the main familial dilated cardiomyopathy (FDCM) related gene target proteins (α/β-Myosin heavy chain, lamin A/C, cardiac actin, etc.) with a frequency of >5%, precluding MR analysis of these etiologies.

## 5 Conclusions

In summary, this study employs MR analysis to examine potential etiological factors of DCM and the causal relationship between myocardial-related proteins and DCM. It was found that alcoholism, perinatal factors, lupus erythematosus, Behçet's disease, hyperthyroidism and thyrotoxicosis, hypothyroidism, and carnitine metabolic disorder have no significant causal relationship with DCM. A causal relationship exists between myocardial-related proteins and DCM, among which titin protein is a protective factor for DCM, whereas cardiac troponin I and desmocollin-2 are risk factors. The application of myocardial cell proteomics in DCM offers promising prospects in the understanding of DCM progression at the molecular and cellular pathway levels (28), facilitating earlier diagnosis of DCM. This also uncovers new molecular therapeutic targets, providing innovative approaches for the treatment of DCM.

## Data Availability

The datasets presented in this study can be found in online repositories. The names of the repository/repositories and accession number(s) can be found in the article/Supplementary Material.
