# Supplementary material for: Exploring the etiology of dilated cardiomyopathy using Mendelian randomization
Source: Front Cardiovasc Med. 2024 Aug 26;11:1364126. doi: 10.3389/fcvm.2024.1364126 (PMC11381399; doi:10.3389/fcvm.2024.1364126)
Supplement: Supplementary file 4 [file Table4.pdf]

Supplementary Table 4

exposure	SNP	effect allele	other allele	β	EAF	SE	P	F
Titin	rs11048398	A	G	-0.2121	0.0905	0.0448	2.19E-06	22.41430664
	rs111515907	G	A	0.1257	0.38525	0.0268	2.82E-06	21.99890009
	rs151177660	A	G	0.4912	0.01648	0.1063	3.80E-06	21.35257162
	rs180853197	T	C	-0.5939	0.01175	0.1233	1.45E-06	23.20066843
	rs220825	A	G	-0.1291	0.31562	0.028	4.17E-06	21.25868622
	rs2293621	A	C	0.2618	0.04924	0.0564	3.55E-06	21.54671546
	rs28454640	G	T	-0.7	0.00673	0.1523	4.27E-06	21.12497839
	rs4696154	G	A	0.1274	0.60261	0.026	1.00E-06	24.01
	rs60997868	T	C	-0.2081	0.1002	0.0438	2.00E-06	22.57334605
	rs73040031	T	C	-0.2615	0.0577	0.0543	1.45E-06	23.19229504
	rs7712746	A	C	0.1946	0.88464	0.0399	1.12E-06	23.78701139
	rs80312778	C	T	-0.3376	0.03001	0.0728	3.47E-06	21.50513223
CTnI	rs1001512	A	G	0.2658	0.05532	0.0547	1.17E-06	23.61213734
	rs10418046	G	T	-0.1419	0.21727	0.0299	2.04E-06	22.52280176
	rs12043231	A	G	0.1409	0.24737	0.0284	6.76E-07	24.61417625
	rs141036107	A	C	0.5758	0.01172	0.1234	3.09E-06	21.77273575
	rs2950828	A	G	0.1664	0.1577	0.0349	1.82E-06	22.73294965
	rs6859877	C	T	-0.1116	0.48075	0.0244	4.79E-06	20.91937651
	rs72829121	C	A	0.188	0.15201	0.0351	8.71E-08	28.68807883
	rs7927835	C	T	0.3319	0.03354	0.0693	1.70E-06	22.9376032
	rs9689951	G	T	0.1169	0.39102	0.0255	4.37E-06	21.01593233
Desmocollin-2	rs11594334	C	T	0.1453	0.20921	2.51E-06	0.0309	3346031265
	rs11960589	A	G	-0.1966	0.10729	1.62E-06	0.041	14694915028
	rs2938171	G	A	-0.1318	0.24903	4.17E-06	0.0286	999612364.8
	rs34001022	C	T	0.3267	0.03084	4.37E-06	0.0712	5601417387
	rs41286884	A	G	-0.4958	0.0135	4.68E-06	0.1082	11236040792
	rs62143198	A	G	-0.1403	0.21453	4.68E-06	0.0306	899737049.8
	rs72672624	T	C	-0.2395	0.07045	6.03E-07	0.048	1.57983E+11
	rs77425280	C	T	-0.3474	0.03385	1.70E-06	0.0726	41846727055
	rs868092	G	A	0.135	0.7395	1.55E-06	0.0281	7597411343
	rs9457933	T	C	0.1454	0.18226	4.47E-06	0.0317	1059566015
perinatal period	rs114420259	A	G	1.406	0.02332	0.303	3.47E-06	21.53205023
	rs16872148	C	T	0.4727	0.358	0.1006	2.63E-06	22.0787887
	rs34113380	A	G	0.5506	0.2098	0.1167	2.38E-06	22.26028406
	rs79722995	G	A	0.9465	0.08289	0.1881	4.88E-07	25.31999674
	rs989625	T	C	-0.7419	0.8844	0.1524	1.13E-06	23.69849727
alcohol	rs117995618	G	A	2.6101	0.009058	0.51	3.09E-07	26.19231838
	rs150882236	C	T	1.2917	0.02435	0.2661	1.21E-06	23.56316205

	rs1574139	C	T	-0.3578	0.3873	0.075	1.85E-06	22.75926044
	rs72732544	C	T	4.1353	0.003402	0.8877	3.19E-06	21.70109275
	rs73144669	G	A	0.4783	0.1495	0.1046	4.83E-06	20.90919884
systemic lupus erythematosus	rs1131114	C	T	0.4233	0.179164	0.0678	4.36E-10	38.97957945
	rs17849502	T	G	0.7862	0.048294	0.14	1.95E-08	31.53624694
	rs3021302	C	T	0.6262	0.141593	0.0724	5.25E-18	74.80816367
	rs4274624	T	C	-0.3778	0.74417	0.0529	9.37E-13	51.00497783
	rs9494895	T	C	0.6663	0.0398049	0.1193	2.31E-08	31.19311449
Behcet's disease	rs1027200	A	G	-0.7519	0.512904	0.1439	1.75E-07	27.30225897
	rs114852128	A	G	1.643	0.0502608	0.3455	1.98E-06	22.61408517
	rs11888082	G	A	1.4937	0.105087	0.3161	2.30E-06	22.32943685
	rs17131954	C	A	0.9002	0.0896474	0.1958	4.28E-06	21.13745238
	rs62463013	A	C	0.9414	0.255725	0.1925	1.00E-06	23.91591054
	rs72735844	T	G	10.2755	0.0121815	2.1409	1.59E-06	23.03632319
hyperthyroidism and thyrotoxicosis	rs10087240	T	C	0.00113355	0.458311	0.000179543	2.30E-10	39.86065285
	rs11736377	T	C	-0.00114545	0.737388	0.00020256	1.40E-08	31.97752863
	rs12741781	G	T	0.00114786	0.328488	0.000190654	1.90E-09	36.24816294
	rs1559810	A	C	0.00107991	0.406014	0.000182147	2.20E-09	35.15046545
	rs1611236	A	G	-0.00105712	0.674823	0.000191225	1.80E-09	30.56041445
	rs163315	T	G	0.00142463	0.145897	0.000253682	2.30E-08	31.53732583
	rs1794279	T	G	0.00688433	0.120389	0.000276549	6.90E-134	619.6966883
	rs3087243	A	G	-0.00192714	0.448193	0.000179831	9.00E-27	114.8411183
	rs3093546	A	G	-0.00237295	0.045312	0.000429397	9.60E-09	30.53930596
	rs3128931	A	G	-0.00119531	0.251339	0.000206247	3.00E-09	33.58812999
	rs41315816	C	T	-0.00244941	0.061221	0.000372226	2.00E-08	43.30216021
	rs4409785	C	T	0.00144029	0.171039	0.000237119	1.10E-09	36.89497167
	rs6679677	A	C	0.00256477	0.096716	0.000301726	1.60E-17	72.25557802
	rs71542456	G	A	0.00413607	0.200752	0.000280011	1.10E-47	218.1853443
	rs9264277	C	T	0.00140179	0.634299	0.000185332	3.00E-17	57.20909665
hypothyroidism	rs10075764	G	A	-0.057	0.302413	0.0104	4.26E-08	30.03883136
	rs10126000	A	C	-0.0683	0.688692	0.0104	5.13E-11	43.12953033
	rs10424978	A	C	-0.0775	0.624328	0.0102	2.77E-14	57.73019992
	rs1079418	G	A	-0.0657	0.262374	0.011	2.14E-09	35.67347107
	rs10917477	G	A	0.064	0.386901	0.01	1.75E-10	40.96
	rs11171710	A	G	-0.0698	0.443916	0.01	3.19E-12	48.7204
	rs11406335	TG	T	-0.057	0.417169	0.0103	3.44E-08	30.62494109
	rs11420448	GT	G	0.1533	0.0467006	0.0275	2.39E-08	31.07555702
	rs11675342	T	C	0.0906	0.387729	0.01	1.40E-19	82.0836
	rs11875260	G	A	0.0751	0.172925	0.0135	2.54E-08	30.94655693
	rs12117927	A	C	0.0627	0.476541	0.0105	2.29E-09	35.65795918
	rs12379417	A	G	0.0583	0.317559	0.0103	1.51E-08	32.0377981
	rs12582330	T	G	-0.061	0.624365	0.0109	2.05E-08	31.31891255
	rs12593201	A	G	0.0905	0.325432	0.0112	7.69E-16	65.29217156
	rs12984428	A	G	-0.0659	0.35604	0.0102	1.11E-10	41.74173395

	rs13090803	T	G	0.0829	0.190624	0.0128	9.00E-11	41.94586182
	rs13109179	A	G	0.0647	0.461895	0.01	9.42E-11	41.8609
	rs1364450	C	A	0.0886	0.126235	0.0139	1.97E-10	40.62915998
	rs141232332	G	GTTT	0.2243	0.451125	0.0127	4.48E-70	311.9256619
	rs142997491	G	A	0.2385	0.0135637	0.0412	7.02E-09	33.5106101
	rs1432806	G	A	0.0583	0.338619	0.0105	2.89E-08	30.82893424
	rs1479565	A	G	0.0975	0.498866	0.0101	7.53E-22	93.1893932
	rs1534430	T	C	-0.086	0.428394	0.0101	1.44E-17	72.50269581
	rs2111485	G	A	0.0813	0.479023	0.0102	1.43E-15	63.53027682
	rs2234167	A	G	0.0825	0.102013	0.015	3.75E-08	30.25
	rs2247314	C	T	-0.086	0.376495	0.0104	1.06E-16	68.38017751
	rs229528	T	C	0.0903	0.496016	0.01	2.31E-19	81.5409
	rs2445608	A	G	-0.0593	0.430366	0.0101	3.79E-09	34.47201255
	rs244685	G	T	-0.0858	0.79915	0.0132	7.06E-11	42.25
	rs28418426	C	T	0.1877	0.501169	0.0133	2.21E-45	199.1706145
	rs2988277	T	C	0.0593	0.287022	0.0106	2.49E-08	31.29663581
	rs307558	A	G	-0.0688	0.742625	0.0119	8.01E-09	33.425888
	rs3087243	A	G	-0.1466	0.386088	0.0102	4.77E-47	206.5701653
	rs3184504	C	T	-0.1734	0.668513	0.0102	7.50E-65	289
	rs3775291	T	C	-0.0649	0.287788	0.0108	1.65E-09	36.11119684
	rs434294	G	A	-0.0683	0.322802	0.0109	3.36E-10	39.26344584
	rs4409785	C	T	0.1069	0.142566	0.0133	8.04E-16	64.60291707
	rs4529854	T	C	-0.0768	0.723438	0.0107	6.56E-13	51.51751245
	rs4835534	C	T	-0.1421	0.156356	0.0132	7.06E-27	115.8884871
	rs5912815	G	T	-0.0511	0.576761	0.0084	1.05E-09	37.00694444
	rs61759532	T	C	0.0905	0.188941	0.0122	1.42E-13	55.02721043
	rs61877856	T	C	-0.0658	0.197495	0.0115	1.14E-08	32.73829868
	rs6679677	A	C	0.3637	0.108444	0.0159	2.39E-115	523.2296586
	rs6908626	T	G	0.1441	0.17075	0.0141	2.04E-24	104.4455007
	rs7030280	T	C	0.2075	0.745664	0.0108	1.02E-82	369.1379458
	rs71508903	T	C	0.0934	0.210816	0.0125	9.34E-14	55.830784
	rs7223956	C	T	-0.0902	0.897005	0.0144	4.27E-10	39.23630401
	rs73192661	T	C	-0.1061	0.428858	0.01	4.05E-26	112.5721
	rs736374	A	G	0.0832	0.375103	0.0103	6.00E-16	65.24875106
	rs7441808	G	A	0.0766	0.21091	0.0111	5.17E-12	47.62243324
	rs7488011	T	C	0.1052	0.356485	0.0111	2.52E-21	89.82257934
	rs7574865	G	T	-0.1321	0.74252	0.0117	1.67E-29	127.4776098
	rs7742626	C	T	0.0686	0.267343	0.0116	3.41E-09	34.97294887
	rs78765971	G	GAC	0.2444	0.146942	0.0162	1.68E-51	227.600061
	rs79490353	C	T	0.2006	0.0241151	0.0349	8.82E-09	33.03779115
	rs7990020	C	A	0.0577	0.453482	0.0101	9.97E-09	32.63689834
	rs853305	C	T	-0.0802	0.718894	0.0111	4.40E-13	52.20387956
	rs881858	A	G	0.0665	0.747899	0.0108	8.46E-10	37.91366598
	rs911760	A	C	0.0879	0.212143	0.0125	1.95E-12	49.449024

	rs926103	C	T	-0.0678	0.697985	0.0104	7.65E-11	42.50036982
	rs9264277	C	T	-0.0862	0.645071	0.0111	9.03E-15	60.30711793
	rs9271365	G	T	0.2484	0.443466	0.0105	4.91E-123	559.6604082
	rs9273371	T	C	0.0791	0.193385	0.014	1.65E-08	31.9225
	rs9277559	C	T	-0.133	0.331026	0.012	1.86E-28	122.8402778
	rs9497965	T	C	0.0827	0.409035	0.0102	3.71E-16	65.73712034
	rs9511151	A	G	-0.0976	0.279567	0.0106	3.08E-20	84.77892488
	rs9902341	T	C	0.0801	0.172257	0.0129	4.68E-10	38.55543537
Carnitine	rs10821585	A	G	-0.0086	0.4124	1.28E-21	9.00E-04	91.30864198
	rs11183620	G	A	-0.005	0.5324	3.00E-08	9.00E-04	30.86419753
	rs11620955	G	A	0.0051	0.5233	1.63E-08	9.00E-04	32.11111111
	rs11620973	G	A	0.0051	0.3674	2.35E-08	9.00E-04	32.11111111
	rs12356193	G	A	-0.0274	0.1638	3.69E-63	0.0016	293.265625
	rs12709393	G	T	-0.0053	0.3714	6.41E-09	9.00E-04	34.67901235
	rs1466788	G	A	0.0074	0.5932	3.05E-16	9.00E-04	67.60493827
	rs2114713	G	T	0.005	0.42	2.67E-08	9.00E-04	30.86419753
	rs2279014	T	C	-0.005	0.367	4.86E-08	9.00E-04	30.86419753
	rs2396004	G	A	-0.0049	0.5611	4.31E-08	9.00E-04	29.64197531
	rs3736438	G	T	0.0057	0.6111	3.19E-10	9.00E-04	40.11111111
	rs419291	C	T	-0.0079	0.6333	3.10E-18	9.00E-04	77.04938272
	rs4860022	T	C	-0.0055	0.3362	9.60E-10	9.00E-04	37.34567901
	rs6862024	A	G	-0.0055	0.3691	8.99E-10	9.00E-04	37.34567901
	rs735315	C	T	-0.0054	0.5057	1.87E-09	9.00E-04	36
	rs9842133	C	T	-0.0064	0.3351	4.20E-12	9.00E-04	50.56790123
renal insufficiency	rs11746266	A	G	-0.071	0.632169	0.0149	1.78599E-06	1580369544
	rs11749282	G	A	-0.0705	0.299804	0.0154	4.84005E-06	212167343
	rs12576766	T	C	0.0964	0.341269	0.0192	4.84195E-07	39638158229
	rs141382820	T	C	0.4636	0.0068660	0.0991	2.87998E-06	25912416221
	rs146184004	T	C	0.287	0.0233833	0.0543	1.269E-07	5.11494E+12
	rs17441789	C	T	-0.1119	0.124408	0.0241	3.58996E-06	971585341.3
	rs185200	G	A	0.0799	0.44108	0.0162	8.19898E-07	9496725927
	rs35129076	T	C	-0.0791	0.236209	0.0169	2.84099E-06	775199630.2
	rs36212726	C	T	0.2424	0.0246191	0.0513	2.32402E-06	10878913125
	rs62006382	G	A	0.105	0.116709	0.0216	0.000001199	7669026385
	rs670501	C	T	-0.0848	0.795931	0.0174	1.06299E-06	6364046535
	rs72867954	C	T	0.3159	0.0140703	0.0682	3.60803E-06	7665826257
	rs7327855	T	G	0.0708	0.359319	0.0154	4.17696E-06	287306785.2
	rs77924615	A	G	-0.1575	0.207812	0.0176	3.8699E-19	1.65639E+35
